# Urban-Rural Differences in Physical Fitness and Out-of-School Physical Activity for Primary School Students: A County-Level Comparison in Western China

**DOI:** 10.3390/ijerph182010813

**Published:** 2021-10-14

**Authors:** Yunxi Tian, Lingfang Liu, Xuhui Wang, Xue Zhang, Yang Zhai, Kai Wang, Jianjun Liu

**Affiliations:** College of Landscape Architecture and Art, Northwest A&F University, Yangling, Xianyang 712100, China; yunxitian@nwafu.edu.cn (Y.T.); llfang0903@nwafu.edu.cn (L.L.); waxx1005@163.com (X.W.); darrrcy@yeah.net (X.Z.); 15559919623@163.com (Y.Z.)

**Keywords:** physical fitness, out-of-school physical activity, urban-rural differences, primary school students, western China, county-level

## Abstract

Rapid urbanization of China has brought lifestyle changes resulting in a continuous decline in children’s physical fitness (PF) and out-of-school physical activity (PA). To date, studies have been focused on correlates of PF and out-of-school PA, and patterns and trends based on geographic diversity and urban-rural contrasts. Western China, with a large rural population, has substantial urban-rural differences, but little work has been done to compare its children’s physical fitness (PF) and out-of-school physical activity (PA) at a county level. A total of 715 primary school students (grades 3–6) were surveyed from one urban school (*n* = 438) and four rural schools (*n* = 277) in a county-level administrative unit, Yangling District, Shaanxi, in western China. Physical fitness index (PFI) was measured and calculated based on the revised Chinese Student Physical Fitness Standards. Out-of-school PA and other variables of demographics, behavior and perception were collected using questionnaires. Statistical analyses explored urban-rural differences and correlates of PFI and out-of-school PA. We found that the PFI (72.86 vs. 79.67) and weekly moderate-to-vigorous physical activity (MVPA) duration (167.57 vs. 220.08) of urban students were significantly lower than those of rural students. Weekly MVPA duration had the largest positive impact on PFI. Perceived availability of PA spaces was positively associated with both the urban and rural students’ PF and PA, while screen time was negatively associated with PF and PA, especially for rural students. Facilitators of PA frequency include the perceived availability of PA time and parental educational level. Parents’ PA habits had a positive impact on urban students’ PA. No association between active school commuting and PF or PA was found. Our findings revealed that PF and out-of-school PA of urban students were clearly lower than among rural students. The health of rural children at the county level in western China should be paid much more attention during the process of rapid urbanization.

## 1. Introduction

Physical fitness (PF) is considered one of the most powerful health markers for children [1,2]. Poor PF scores are related to increases in cardiovascular disease risk, type 2 diabetes, hypertension, stroke and mortality [3]. Next to genetic factors, physical activity (PA) is considered the most manageable factor influencing PF [4,5], especially for children with relatively fixed patterns of lifestyle behaviors and living environments. Indeed, the health benefits of PA for children have been well documented [6,7,8], especially the outdoor PA. Regular participation in PA can form a lifelong exercise habit for children and result in a decreased risk of obesity and low bone density among children. Moreover, outdoor PA also provides opportunities for children to socialize with friends, interact with nature and obtain mental health benefits.

However, the PA and PF trends in children have not been positive in recent decades [9]. Globally, more than 80% of students aged 11–17 years do not meet the recommended PA level set by the World Health Organization, especially students in the high-income countries in the Asia-Pacific region [10]. In China, rapid urbanization with economic development has brought changes in lifestyles, such as the prevalence of high-fat foods and smart devices for young people [3,11]. Only about one third of school children (grades 1–12) can meet the recommended PA level, and 30% of school children can achieve a “good” or “excellent” rating of PF [12], which are lower than some developed countries, e.g., Slovenia [13]. Si et al. [14] estimated that only 8% of school children participate in out-of-school moderate or vigorous PA. Meanwhile, the excessive use of smart devices contributes to children’s sedentary lifestyles [15]; over 75% of Chinese young people have more than 2 h of daily sedentary behavior time [16].

Improving children’s PF and out-of-school PA has been an academic research focus in fields of children’s health [17,18], physical education [3,19] and built environments [20,21]. Relevant studies have been conducted from largely two perspectives, correlates (or associated factors, barriers or facilitators) of out-of-school PA, and patterns and trends based on geographic diversity and urban-rural differences [17,19,20,22]. The potential barriers included screen time [20,22,23], unattractive facilities and parental restrictions due to safety concerns [20]. Facilitators were found to include active school commuting [24], accessibility of facilities [19], perception of availability of more parks [21], parental educational level [25], and frequency or duration of parents’ PA with children [26]. These correlates of out-of-school PA can be summarized into environmental, personal and family, or behavioral domains. Surprisingly, few studies have investigated the correlates of PF.

PA and PF are interconnected, but different concepts. PA is defined as “a behavior involving movement of the body through space” and PF refers to a state or a condition related to the ability to perform PA and covers a full range of physiological qualities [3,27]. Existing studies often assume that PA contributes to the improvement of PF, yet PA plays a small role in affecting some indicators of PF [5,27]. A practical solution is to simultaneously consider the influence of both PA and PF factors on children’s health.

There is evidence supporting the significance of urban-rural differences in children’s PF and out-of-school PA [19,28,29]. Findings to date appear to lack consistency among studies conducted in different countries or regions [17,28,29,30]. Huang et al. [19] suggested that much more study of children’s PA is needed within a socioecological framework related to geographical differences combined with other socioeconomic factors. In densely populated China as a whole, there are urban-rural differences in the PF of school-aged children [17], and the diversity of internal socioeconomic landscapes tend to imply that the urban-rural differences in western inland and eastern coastal regions should show considerable variation. On one hand, rural areas in eastern China have been highly industrialized and urbanized since the Chinese economic reform or reform and opening-up in the 1980s. Since then, urban-rural differences have been gradually lessened, or even eliminated in some cases. For example, one study conducted in Jiangsu Province in eastern China found that urban and rural students had no significant differences in PA level [18]. However, central and western China, with 70% of the country’s rural areas, had substantial urban-rural differences, as 70% of the population is still engaged in traditional agriculture [31]. Studies have shown that rural areas in western China have much lower levels of sport resource allocation, and their residents pay less attention to health and PA compared to those in eastern China [32,33,34].

This suggests to us that western China provides ideal geographical and socioeconomic context to investigate urban-rural differences in PF and out-of-school PA for children and adolescents. Compared to metropolitan cities (e.g., Chile’s Valparaíso [35], Mexico’s Guadalajara [36], Taiwan’s Taipei [19] and China’s Shanghai [37]), urban and rural areas within county-level administrative units are much closer geographically and residents are likely to possess more similarities and connections on the basis of biological characteristics and culture. In this way, correlates of children’s PF and out-of-school PA become more statistically comparable, and conclusions can be more justifiably generalized with targeted interventions for improving children’s PF and out-of-school PA.

In summary, the limitations and necessities of research on the urban-rural differences in children’s PF and out-of-school PA at a county level in western China call for a more detailed analysis. This study aimed to fill the knowledge gap by investigating differences in out-of-school PA patterns and correlates of PF and out-of-school PA for primary school students in urban and rural areas of Shaanxi province, China. Therefore, the objectives of this study were: (1) to examine urban-rural differences in PF and out-of-school PA for primary school students at a county level in western China, and (2) to explore the associations between demographics, behavior, perception, parents’ educational level and PA habits, and PF as well as out-of-school PA, and (3) to examine the urban-rural differences among the above associations.

## 2. Materials and Methods

### 2.1. Study Area

Data were collected in Yangling agricultural high-tech industry demonstration zone (referred to as Yangling District). This is a county-level administrative unit, located in the central area of Shaanxi Province, and the surrounding villages and towns are mostly located in the mountains. Yangling District had 16 square kilometers of total built-up area and a total population of 212,300 as of December 2019, of which 31,850 were students in primary and secondary schools. The per capita GDP in Yangling District was 79,115 Yuan in 2019, ranking it third in Shaanxi Province [38].

### 2.2. Study Sample and Design

The sample consisted of 731 primary school students aged 8–13 enrolled in the third to sixth grades. Seven hundred and fifteen students provided complete data for the analysis with a response rate of 97.8%. Students came from one urban school (*n* = 438) and four rural schools (*n* = 277). To obtain representative survey data, school selection was based on the diverse geographic areas in Yangling District. The urban school was the Yangling High-tech Primary School, which has the largest number of students in the area. Two classes of third to sixth grade students in the urban school were randomly selected for participation. The four rural schools were selected from four typical mountainous villages. All classes in the third to sixth grades in the four rural schools were selected for participation, and all students in these classes were enrolled in this study. The sample proportion of students from urban and rural primary schools was consistent with the proportion of urban and rural population at the end of 2019 in Shaanxi Province [39].

Informed consent was obtained from students, their guardians, school administrators and teachers prior to participation. The goals, procedures, questionnaires and PF test in the study were carefully explained to the students by their teachers during class time, and students were assured that their responses would remain confidential. Each student was assigned a number in advance, and their responses were anonymous and independent. The completion time of the questionnaire was controlled to be within 5 min. After completing the questionnaire, students went to the corresponding venue to perform the PF tests required by the study.

### 2.3. Measurements

The survey focused on the measurement of the level of students’ out-of-school PA and their PF. Throughout the survey process, all measurements were taken at schools during school hours. The data collection period lasted from June to July 2020.

#### 2.3.1. Out-of-School Physical Activity

Out-of-school PA (hereinafter referred to as “PA”) in the study is defined as children’s self-reported participation in outdoor activities after school, excluding school physical education. The PA questionnaire was simplified based on the short form of the International Physical Activity Questionnaire (IPAQ) to quantify the level of PA undertaken by students. IPAQ was previously used to assess students’ PA and had well-established validity [8,19]. The PA questionnaire included a total of four questions for frequency and duration of moderate physical activity (MPA) and vigorous physical activity (VPA): “In the past 7 days, how often did you engage in MPA/VPA?” and “How long did you spend on MPA/VPA on average each time?”. Considering primary school students’ comprehension, we interpreted MPA in the question as “After MPA, I feel a bit tired, though, I can breathe normally, such as water play, walking and gardening, etc.”; we interpreted VPA as “After VPA, I’m sweating, breathing a lot, and I feel tired, such as ball games, running and jumping chases, roller-skating and other games”. Students were asked to report in each category the frequency (none, once a week, 2–3 times a week, 4–5 times a week, 6–7 times a week, or more than 7 times a week) in the past 7 days and the average duration of time spent on each occasion (less than 15 min, 15–30 min, 30–60 min, 1–2 h, or more than 2 h). All questions were single choice.

To facilitate quantitative comparison of PA levels between urban and rural students, the frequency and average duration of students’ PA were converted to the weekly moderate-to-vigorous physical activity (MVPA) duration (a continuous variable). The options describing the frequency in the past 7 days (none, once, 2–3 times, 4–5 times, 6–7 times, or more than 7 times) were respectively converted to the values “0”, “1”, “2.5”, “4.5”, “6.5” and “8” times/week. The options describing the average duration of time spent each time (less than 15 min, 15–30 min, 30–60 min, 1–2 h, or more than 2 h) were respectively converted to the values “7.5”, “22.5”, “45”, “90” and “180” min per time. Weekly MVPA duration was calculated using a formula, i.e., weekly MVPA duration = MPA frequency (times per week) × MPA duration (min per time) + VPA frequency (times per week) × VPA duration (min per time).

The second questionnaire comprised sections that were generated based on previous studies [19,40], and was presented in three parts. The first part referred to students’ and their parents’ demographics (i.e., sex, age, grade, parents’ educational level, and parental PA habits). The second part asked for information on commuting to and from school (hereafter referred to as “the commute”) and the average daily screen time (less than 30 min, 0.5–1h, 1–2 h, 2–3 h, and more than 3 h; hereafter referred to as “screen time”). The commute included modes of transport to and from school (i.e., walking, cycling, public transit or private car) and the length, in time, of the commute (less than 5 min, 5–15 min and more than 15 min). In the third section of the questionnaire, students were asked what activities they participated in after school (i.e., homework, watching TV or playing electronic devices, engaging in outdoor PA, or going to cram school (preparation for examinations), the time and place (i.e., park or woodland around the village, community open spaces or farmland, yard, city or village square, city or village road, stadium, scenic area and other) of PA on school days and non-school days, and the perceived availability of PA spaces and time (using the items “There are a lot of usable PA spaces in my neighborhood” and “ There is a lot of time for PA” with a 5-point scale, from 1 “strongly disagree” to 5 “strongly agree”; hereafter referred to as “perceived availability of PA spaces or time”). For their responses to questions regarding PA time and place, respondents could select multiple options; all other questions were single choice.

#### 2.3.2. Physical Fitness

Children’s physical fitness levels were quantified in a physical fitness index (PFI) using the revised 2014 version of the Chinese Student Physical Fitness Standards (hereinafter referred to as “Standards”) [41]. PFI was calculated by scoring indicators of body shape, body function and body quality (strength and endurance), weighted as 15%, 15% and 70%, respectively (Table 1). Body quality tests were performed only once because they were physically challenging. The measured value of each indicator was converted into scores based on grades and gender according to the Standards. The children’s PF test is a task that all schools need to perform annually, and all normal students are covered. The single PF test took 1–2 days for each grade, and the overall testing session in our study lasted one month. The testing included three parts. First, students in a group of 4 were organized to test weight, height and vital capacity. Second, students in a group of 4 were arranged to test one-minute sit-up and one-minute rope jumping. After a 30-min break, students in a group of 4 were required to perform an 8 × 50 m shuttle run trial. Finally, students in a group of 4 were required to test sit and reach, and afterwards students in a group of 5 were allowed to perform a 50 m dash trial. The PF test was administered by the physical education teachers in each grade. All investigators from the research team received a one-week training session on the use of standardized protocols and instruments for data collection. Before each test, physical education teachers would give a quick demonstration and provide guidance. Good reliability has been reported for all the tests used in the study.

### 2.4. Statistical Analysis

The data analysis process included descriptive statistics, bivariate analyses and regression analysis. Descriptive statistics were first examined to identify students’ preferred PA places and times on school days and non-school days in urban and rural areas. Next, chi-square tests were conducted to investigate differences among categorical variables between urban and rural areas. For continuous variables, independent-sample *t*-tests were conducted to investigate urban and rural differences. Finally, ordinal logistic regression analysis was performed to explore the association between significantly correlated variables and MPA and VPA frequency. Multiple regression analysis was conducted to examine relationships between significantly correlated variables and PFI.

To meet the assumptions of the linear regression model, all categorical variables were converted to dummy variables according to the number of options, and dummy variables were coded as “1” or “0”, e.g., the variable “commuting time” was converted to dummy variable as “commuting time 1 (1 = less than 5 min, 0 = non-less than 5 min)”, “commuting time 2 (1 = 5–15 min, 0 = non-5–15 min)” and “commuting time 3 (1 = more than 15 min, 0 = non-more than 15 min)”. The independent variables were selected by stepwise regression. To avoid multicollinearity, the commute variables and parents’ demographic variables were excluded. Logistic regression analysis results were presented as an odds ratio (OR) with 95% confidence interval (CI). Linear regression analysis results were presented both as unstandardized and standardized coefficients. We use the Kolmogorov–Smirnov statistic to test for normality in all analyses. All analyses were carried out in SPSS version 26 (IBM Corp, Armonk, NY, USA), and statistical significance was determined at a 0.05 level for all analyses.

## 3. Results

### 3.1. Characteristics of Participants

Among the 715 respondents, 438 (61.3%) were urban and 277 (38.7%) were rural students (Table 2). Of the total, 53.6% of the students were male and 10–11 years old was the main age group (48.7%). Significant differences were found across all key variables between the urban and rural students, except for the students’ gender and age.

The proportion of students living in rural areas who chose public transit for school was significantly higher than those in urban areas (26.4% vs. 10.5%), and more than half of urban students commuted to school in a private car. Urban students usually spent 5–15 min commuting (71.0% vs. 36.1%). The proportion of rural students who spent less than 5 min (21.3% vs. 13.2%) and more than 15 min (42.6% vs. 15.8%) commuting was significantly higher than that of urban students.

Urban (32.9%) and rural (45.5%) students both reported having “less than 30 min” of daily screen time. The proportion of rural students with “more than 3 h” of screen time was significantly higher than that of urban students (6.6% vs. 1.2%), while urban students had a higher proportion of screen time in the 0.5–1 h range. Rural students reported a higher perceived availability of PA space and time. Urban students had significantly lower PFI values (72.86) than rural students (79.67). An 8.6% share of parents in rural areas had a bachelor’s degree or above, but 77.4% of parents in urban areas had a bachelor’s degree or above. More parents in urban areas reported having PA habits (87% vs. 77.3%).

### 3.2. PA Patterns among Urban Students vs. Rural Students

#### 3.2.1. Frequency and Duration of PA

**Figure 1:**
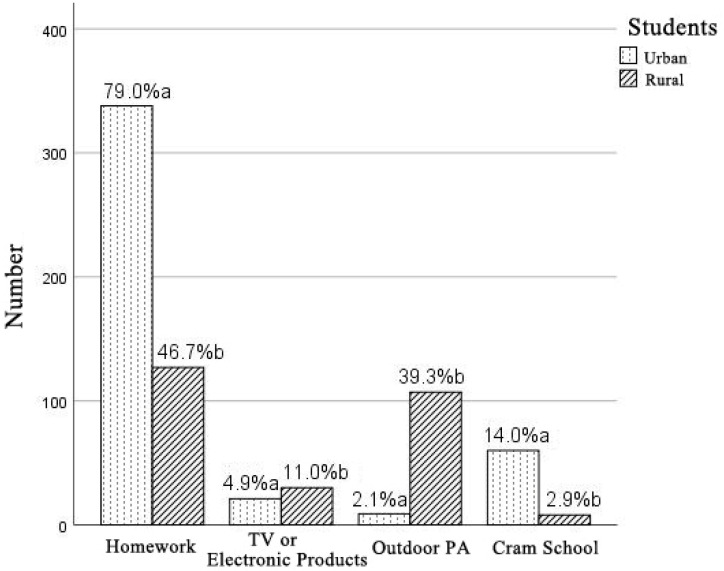
Contrast between urban and rural students in after-school activities. The letters “a” and “b” indicate significant differences (*p* < 0.05).

As seen in Figure 1, 93% of urban students participated in the after-school activities of homework and cram school, and 49.6% of rural students chose homework and cram school. The number of rural students participating in PA was far more than urban students (39.3% vs. 2.1%).

The largest proportion of urban and rural students reported “2–3 times a week” and “15–30 min” MPA and VPA (Table 3). Urban and rural students reported significantly different PA frequency and VPA duration. Compared with the rural students, the urban students had lower “6–7 times a week” MPA (4.8% vs. 10.1%), but higher “once a week” MPA (19.9% vs. 12.3%). The proportion of “none” and “once a week” VPA in urban students were higher than among rural students, and the ratio of “4–5 times a week or above” VPA was lower than that for rural students. Urban students had more “less than 15 min” VPA (36.3% vs. 24.9%), but fewer “30–60 min” VPA (13.7% vs. 20.2%) responses. Overall, the urban students had low MPA and VPA frequency, and significantly lower weekly MVPA duration than rural students (167.57 vs. 220.08).

#### 3.2.2. Place and Time of PA

On school days, 56.8% of urban students chose to engage in PA in community open spaces, followed by parks (37.4%) (Table 4). The frequencies of six other places were below 10%. Yards (51.3%) were the most frequently used spaces for PA among rural students on school days, and squares (31.8%) were the second most common.

On non-school days, community open spaces (33.3%) and parks (24.1%) were also the most frequently used PA spaces among urban students, but their number of reported uses was less than during school days. The proportion of users of the other six places increased on non-school days, especially the scenic areas (22.4% on non-school days vs. 0.9% on school days). Similarly, yards (36.8%) and squares (35.0%) were the most used PA places for rural students, and the number of uses of “yards” decreased dramatically on non-school days, but the proportion of use of other places increased. The overall ranking of places frequently used by rural students was similar between school days and non-school days except woodland. On non-school days, three types of PA spaces (yards, squares and roads) were used by more than 25% of rural students, while only one PA space (community open spaces) was used by more than 25% of urban students.

On school days, 56.7% of urban students chose to engage in PA between 18:00–20:00, followed by 20:00–22:00 (27.3%); proportions of users during other periods were below 20% (Table 5). Three time periods (18:00–20:00, 12:00–14:00 and 5:00–7:00) were frequently used PA times by rural students, of which 18:00–20:00 was used by 40% of rural students. Only 3% of rural students chose to engage in PA after 20:00. On non-school days, all the time periods before 18:00 were used by about 20%–30% of rural students, but the frequency of users decreased after 18:00. Urban students mainly chose to engage in PA between 16:00–18:00 (31.6%) and 18:00–20:00 (48.0%). Overall, the PA time of urban students was mainly after 16:00, but rural students tended to engage in PA before 18:00.

### 3.3. Factors Associated with PA Frequency and PFI of Urban Students vs. Rural Students

#### 3.3.1. Factors Associated with MPA Frequency and VPA Frequency of Urban and Rural Students

The significance of the test of parallel lines and the goodness-of-fit of the six models were over 0.05, and the likelihood ratios were below 0.05, which indicated that the regression models were applicable. Given the large number of explanatory variables examined, Table 6 and Table 7 only present results for those statistically significant factors (*p* < 0.05).

Results from the combined (urban and rural student) samples showed that relative to more than 3 h screen time, students who had less than 30 min of screen time daily were more likely to achieve higher MPA frequency (OR = 3.991, Table 6). Students who perceived an abundance of available PA spaces and time were more likely to have higher MPA frequency in the three models. No other variables were found to be associated with MPA frequency.

Results from combined (urban and rural student) samples showed that students who perceived an abundance of available PA spaces and time were more likely to have higher VPA frequency (Table 7). Perceived availability of PA space was associated with VPA frequency in urban and rural samples.

Compared with those students whose screen time was more than 3 h, students with less than 30 min screen time were more likely to score higher VPA frequency (OR = 2.542, Model 1). Rural students with less than 30 min daily screen time were 3.2 times more likely to achieve higher VPA frequency compared to those with more than 3 h daily screen time. No significant association was found between screen time and VPA frequency in urban students.

Compared to urban students whose parents reported PA habits, urban students whose parents did not have PA habits were less likely to have higher VPA frequency. No significant association was found between parents’ PA habits and VPA frequency in rural students.

Students whose parents had a junior high school or lower educational level were 1.8 times more likely to have high frequency of VPA compared to those whose parents had a graduate degree in the combined samples. No other variables were found to be associated with VPA frequency.

#### 3.3.2. Factors Associated with PFI of Urban and Rural Students

The adjusted R^2^ of the combined, urban and rural samples was 0.510, 0.299 and 0.522, respectively (*p* < 0.001, Table 8). The unstandardized residual mean was zero and the significance of Kolmogorov–Smirnov test results for the three models was greater than 0.05, which indicated that the residuals satisfied a normal distribution. The variance inflation factor of the independent variables of the three models was between 1 and 1.2, indicating that multicollinearity could be neglected.

For combined samples (Model 1), weekly MVPA duration had the largest impact on PFI (beta = 0.403) and was positively associated with PFI. The degree of influence of other variables on PFI in descending order was urban area, perceived availability of PA space and screen time, all of which had a negative influence except for perceived availability of PA space. No other significant relationships were found.

Factors associated with PFI in urban and rural samples were slightly different. Weekly MVPA duration had the largest impact on PFI in both groups (Model 1 and Model 2), but the impact was larger in rural samples (Model 3). Only daily screen time of more than 3 h had a negative impact on the PFI of rural students. Perceived availability of PA space was positively associated with PFI in urban and rural samples. No other significant relationships were found.

## 4. Discussion

The findings from this study showed that the PFI among urban students was significantly lower than that of rural students (Table 2). This was consistent with the findings from the Physical Activity and Fitness in China—The Youth Study [17] that children living in urban areas were less likely to pass the fitness standards (PFI no less than 60.0), compared with those living in rural areas.

The factors affecting PFI included the weekly MVPA duration, perceived availability of PA spaces, and screen time (Table 8). The weekly MVPA duration had the largest positive impact on PFI for both urban and rural students, and urban students had significantly lower weekly MVPA duration than rural students (Table 3). This was consistent with the findings using the different physical fitness measurements in developed countries that vigorous PA was positively associated with the global fitness score (similarly to the PFI in this study) [5]. Our finding agreed with the findings from a study recently conducted in another Chinese city that low PA levels may lead to low physical fitness levels [42]. However, there was disagreement on the comparison of the weekly MVPA duration between urban students and rural students. Moore et al. [30] found that mean daily MVPA duration was significantly higher for urban youth compared to rural youth from three U.S. middle schools. A study from Australia showed no significant differences in daily MVPA duration between urban children and rural children aged 5 to 12 [28]. The reasons for these mixed results may be attributed to the different economic incomes of urban and rural households. A study from Taiwan [19] indicated that rural students came from more affluent families compared to urban students, and their PA levels were lower. However, in our study, the household incomes of rural students were lower, but their PA levels were higher.

Both urban students and rural students who perceived that more PA spaces were available in their surroundings were more likely to have higher PFI (Table 8) as well as MVPA frequency (Table 6 and Table 7). In our study, the perceived availability of PA spaces was significantly lower for urban students compared to rural students (Table 2). A study from the Netherlands had similar findings—that the perception of greater availability of sports facilities was more likely to increase sports participation among adolescents from 12 to 15 years of age [43]. Improving the perceived availability of PA spaces could lead to after-school active lifestyles. Although the perceived availability of PA spaces was usually constrained by the objective availability, some studies found objective facility measures unrelated to PA [44,45]. There may be a mismatch between perceived and objectively assessed environments (e.g., PA spaces), especially among younger and older women [46]. Moreover, the perceived availability of PA spaces can be affected by individual subjective factors. Studies showed that more active people were more likely to notice facilities that provide opportunities for PA [43,44]. The rural students who were more physically active were more sensitive to the available opportunities for PA.

Screen time was another significant factor that was negatively associated with students’ PFI (Table 8) and MVPA frequency (Table 6 and Table 7), which was consistent with studies from other countries [20,47,48]. More screen time contributed to a student sedentary lifestyle [49,50,51] that has adverse health effects such as myopia and obesity [52,53]. The increase in screen time tended to make children lose opportunities to engage in outdoor PA [54], and a rising number of children preferred to interact with their peers through electronic devices rather than outdoor PA [20].

Our findings also indicated that screen time had a greater negative impact on rural students’ VPA frequency (Table 7). According to the Report on Child Development in China, rural children had significantly higher daily screen time than urban children (108 min vs. 88 min) [55]. Thus, rural students’ PA levels are more likely to decline under the influence of increased screen time. Our study also showed that the number of rural students who chose to watch TV or electronic devices after school was significantly higher, which may be due to the limited out-of-school lifestyle and lack of adult supervision or family rules in rural areas. It was inconsistent with findings reported in Australia, for instance, where rural children had less screen time than urban children [28]. The Chinese government has released a series of policies that aim to restrict and reduce student screen time, but these policies are mainly based on the prevention of myopia [56]. Future policies should broaden their scope to include the impact of screen time on students’ PA and PF. Furthermore, our findings highlighted the need for more targeted policies and intervention efforts for rural students, such as organizing collective activities to enrich rural students’ after-school life by their local communities.

Without having a direct impact on PFI, the perceived availability of PA time was one of the significant factors that raised students’ MVPA frequency in this study (Table 6 and Table 7). Similarly, the perceived availability of PA time for urban students was significantly lower than that for rural students (Table 2). The low perceived availability of PA time could result in students being less likely to participate in outdoor PA. An increased emphasis of academic and examination-oriented education in China has de-emphasized PA for students [3,22], removing time available for PA. Especially in urban areas, parents with higher educational levels are likely to place more importance on their children’s academic achievement and enroll their children in various cram schools. Compared to rural students, urban students were under more pressure to succeed academically, so more time was allocated for studying [57,58]. This was consistent with our finding that more urban students chose to do homework and attend cram school classes after school than rural students (93% vs. 49.6%).

Parents have an important influence on children’s PA [57,59]. As Akpinar and Cankurt [26] stated, “physically more active parents bring up physically more active children”. We found that urban students whose parents did not have PA habits were likely to have lower VPA frequency (Table 7). However, the results from rural students did not agree with the conclusion. One possible explanation may be that, compared to urban students, rural students especially in China’s western provinces are separated from their parents for long periods as parents often leave home to work in cities for better incomes [60]. Another influence may be related to the educational level of parents. Findings from European countries showed that the educational level of parents, especially mothers, was positively associated with children’s PA [25,61]. However, a study from Turkey demonstrated that parental education level was negatively associated with children’s PA level [26]. In our study, students whose parents had a junior high school education or less were more likely to have higher VPA frequency. This may infer that other socioeconomic factors (e.g., household incomes and parental occupation) possibly have interactive effects on children’s PA.

Previous studies have shown that active commuting to and from school was correlated with higher PA level and physical well-being [24,62,63]. However, no associations with these variables were confirmed in our study. Rural students were more likely to choose active commuting modes (i.e., walking and cycling) to and from school, compared to urban students. This was consistent with findings from the China Puberty Research Collaboration [63].

To our knowledge, this study was the first investigation of urban-rural differences in PA and PF for primary school students in western China at a county-level. Another important strength was the statistical children population aged 8–13, who have a higher demand for outdoor PA. The primary limitation of this study was that it did not investigate the objective built environments of students’ residential communities. Thus, the study did not cover the relationship between objective built environment and students’ PA and PF. We are aware that self-reported data may bring uncertain bias to study results and that causal relationships cannot be inferred from the cross-sectional study. Despite these limitations, our study contributes to the limited literature on the urban-rural differences in county-level areas in western China, and provides a basis for continued investigations into the interaction of children’s PA and PF. The study also emphasized the importance of paying attention to children’s perceptions in developing intervention programs.

## 5. Conclusions

We found that the PFI and MVPA duration of urban students were significantly lower than those of rural students at a county level in western China. Compared to the national average [64], the normalized average daily MVPA duration for both urban and rural students is much lower (26.8 min/day vs. 45.4 min/day). Due to the targeted urbanization policy, county-level areas in western China are undergoing the most rapid change to urbanization [11,65]. Although urbanization has promoted economic development and enriched available sports facilities, unhealthy lifestyles including high-fat diets and sedentary behaviors have become widespread and accelerated the decline of health indicators in more urbanized areas [11]. The health of rural children in county-level areas in western China should focus much more attention on the adverse effects caused by urbanization. Analyzing urban-rural differences is the primary step in formulating policies and interventions to reduce threats of urbanization to the health of rural children.

Our findings suggest that if the perceived availability of PA space and time are improved, both urban and rural students will likely be more physically active. This creates opportunities for developing intervention programs to focus on the children’s perceptions. Potential intervention strategies include encouraging schools to assign PA as part of homework to students in order to guarantee sufficient time for PA; enhancing health education in schools and society to make students and parents aware of the importance of active lifestyles; and improving the availability of sport facilities for children, e.g., increasing the amount of PA space around the communities, or improving quality in the provision of sport facilities. In addition, parents’ PA habits have a positive effect on out-of-school PA levels of urban students, and screen time has a greater negative impact on that of rural students. Therefore, the involvement of parents, school and society in efforts to improve urban students’ out-of-school PA is recommended, while intervention strategies to promote rural students’ out-of-school PA should concentrate on limiting screen time. Future studies would benefit from investigating the effects of the built neighborhood environment on both urban and rural students’ out-of-school PA levels and PF to underpin stronger support for policymakers and professionals.

## Figures and Tables

**Table 1:** The Measurement Indicators of PFI.

Category	Indicator/Unit	Description and Methods	Number of Measurements
Body Shape	Body Mass Index (BMI)/kg/m^2^	BMI is defined as the body mass divided by the square of the total height.	Weight and height were measured twice, and the mean value was recorded.
Body Function	Vital Capacity (VC)/ml	VC refers to the amount of air that the lungs can expel after having been filled completely, and was measured with spirometry.	The VC was measured twice, and the high value was recorded.
Body Quality	50m Dash/s	To measure acceleration and speed, the test requires students to start at a unified starting point and records the time when students finish the 50 m distance.	The test of each indicator was performed once.
	Sit and Reach (SR)/cm	To measure flexibility, in the sitting position with knees straight and feet flat against the vertical support, students’ hands slide the ruler as far as they can.	
	One-minute Sit-up (SU)/times	To measure abdominal muscular endurance, the test requires students to lay on their back with the knees bent and feet flat on the floor held by a partner. Students’ fingers crossed and held behind the head. During the test, children were asked to perform as many correct sit-ups as possible in one minute test period.	
	One-minute Rope Jumping (RJ)/times	To measure motor coordination, students were asked to jump continuously for one minute and investigators recorded the total number of jumps.	
	8 × 50 m shuttle run/s	To measure flexibility and endurance, students were asked to run back and forth 8 times around the straight track between two poles 0.5 m away from the start line and the finish line. The distance between the start line and the finish line was 50 m.	

**Table 2:** Key Variables of Urban vs. Rural Students: Descriptive Statistics.

Variable		N (%) or Mean ± SD			Sample Difference
		Urban (*n* = 438)	Rural (*n* = 277)	Total (*n* = 715)	
Students’ Characteristics					
Gender	Male	241 (55.0%)	142 (51.3%)	383 (53.6%)	*χ*^2^ = 0.0964 (*p* = 0.326)
	Female	197 (45.0%)	135 (48.7%)	332 (46.4%)	
Age	8–9	104 (23.7%)	54 (19.5%)	158 (22.1%)	*χ*^2^ = 3.512 (*p* = 0.173)
	10–11	216 (49.3%)	132 (47.7%)	348 (48.7%)	
	12–13	118 (26.9%)	91 (32.9%)	209 (29.2%)	
Behavior					
The Commute					
Commuting Style	Walking	157 (35.8%)	111 (40.1%)	268 (37.5%)	*p* < 0.001 ^1^
	Cycling	7 (1.6%)	2 (0.7%)	9 (1.3%)	
	Public transit	46 (10.5%)	73 (26.4%)	119 (16.6%)	
	Private car	228 (52.1%)	91 (32.9%)	319 (44.6%)	
Commuting Time	<5 min	58 (13.2%)	59 (21.3%)	117 (16.4%)	*χ*^2^ = 84.917 (*p* < 0.001)
	5–15 min	311 (71.0%)	100 (36.1%)	411 (57.5%)	
	>15 min	69 (15.8%)	118 (42.6%)	187 (26.2%)	
Screen Time	<30 min	144 (32.9%)	126 (45.5%)	270 (37.8%)	*χ*^2^ = 33.510 (*p* < 0.000)
	0.5–1 h	168 (38.4%)	71 (25.6%)	239 (33.4%)	
	1–2 h	77 (17.6%)	47 (17.0%)	124 (17.3%)	
	2–3 h	44 (10.0%)	15 (5.4%)	59 (8.3%)	
	>3 h	5 (1.2%)	18 (6.6%)	23 (3.2%)	
Perception					
Perceived Availability of PA Spaces		3.25 ± 1.300	3.75 ± 1.232	3.44 ± 1.296	*t* = −5.132 (*p* < 0.001)
Perceived Availability of PA Time		3.00 ± 1.279	3.69 ± 1.250	3.27 ± 1.311	*t* = −7.056 (*p* < 0.001)
Physical fitness					
PFI		72.86 ± 7.87	79.67 ± 5.76	75.50 ± 7.86	*t* = −13.324 (*p* < 0.001)
Parents’ Characteristics					
Highest Educational Level	Junior High School or Less	27 (6.2%)	164 (59.2%)	191 (26.7%)	*χ*^2^ = 356.258 (*p* < 0.001)
	High School	72 (16.4%)	89 (32.1%)	161 (22.5%)	
	Bachelor’s Degree	212 (48.4%)	20 (7.2%)	232 (32.4%)	
	Graduate Degree	127 (29.0%)	4 (1.4%)	131 (18.3%)	
Have PA Habits		381 (87.0%)	214 (77.3%)	595 (83.2%)	*χ*^2^ = 11.502 (*p* = 0.001)

^1^ Fisher’s Exact Test.

**Table 3:** Comparison of Urban and Rural Student Frequency and Duration of PA.

Variables		Urban (*n* = 438)		Rural (*n* = 277)		Sample Difference	
		MPA	VPA	MPA	VPA	MPA	VPA
Frequency	None	7.8%	12.1%	7.6%	5.4%	*χ*^2^ = 14.297 (*p* = 0.014)	*χ*^2^ = 75.687 (*p* < 0.000)
	Once a week	19.9%	34.2%	12.3%	17.7%		
	2–3 times a week	44.5%	39.0%	42.6%	37.9%		
	4–5 times a week	20.3%	11.2%	23.8%	20.9%		
	6–7 times a week	4.8%	2.3%	10.1%	13.4%		
	>7 times a week	2.7%	1.1%	3.6%	4.7%		
Duration	Less than 15 min	17.1%	36.3%	16.6%	24.9%	*χ*^2^ = 3.985 (*p* = 0.396)	*χ*^2^ = 13.241 (*p* = 0.008)
	15–30 min	40.6%	42.0%	47.7%	48.0%		
	30–60 min	31.1%	13.7%	27.1%	20.2%		
	1–2 h	10.7%	7.3%	8.3%	6.1%		
	More than 2 h	0.5%	0.7%	0.4%	0.7%		
Weekly MVPA duration		167.57 ± 161.33		220.08 ± 228.96		*t* = −3.330 (*p* < 0.001)	

**Table 4:** PA Places Where Students Played on School Days and Non-school Days.

PA Places ^1^	School Days				Non-School Days			
	Urban (*n* = 438)		Rural (*n* = 277)		Urban (*n* = 438)		Rural (*n* = 277)	
	Frequency (%)	Rank	Frequency (%)	Rank	Frequency (%)	Rank	Frequency (%)	Rank
Parks/Woodland	164 (37.4%)	2	23 (8.3%)	5	105 (24.1%)	2	24 (8.7%)	7
Community open spaces/Farmland	249 (56.8%)	1	36 (13.0%)	3	146 (33.3%)	1	48 (17.3%)	4
Yards	16 (3.7%)	4	142 (51.3%)	1	31 (7.1%)	6	102 (36.8%)	1
Squares	11 (2.5%)	6	88 (31.8%)	2	40 (9.1%)	5	97 (35.0%)	2
Roads	14 (3.2%)	5	34 (12.3%)	4	21 (4.8%)	7	70 (25.3%)	3
Stadiums	25 (5.7%)	3	23 (8.3%)	5	48 (11.0%)	4	38 (13.7%)	5
Scenic areas	4 (0.9%)	7	5 (1.8%)	6	98 (22.4%)	3	27 (9.7%)	6
Others	0	8	1 (0.4%)	7	0	8	4 (1.4%)	8

^1^ “/” stands for urban PA place/rural PA place.

**Table 5:** PA Time When Students Played on School and Non-school Days.

PA Time	School Days				PA Time	Non-School Days			
	Urban (*n* = 438)		Rural (*n* = 277)			Urban (*n* = 438)		Rural (*n* = 277)	
	Frequency (%)	Rank	Frequency (%)	Rank		Frequency (%)	Rank	Frequency (%)	Rank
5:00–7:00	72 (18.9%)	3	102 (37.8%)	3	6:00–8:00	63 (14.5%)	6	60 (21.7%)	4
12:00–14:00	38 (10.0%)	4	103 (38.1%)	2	8:00–10:00	73 (16.9%)	5	80 (28.9%)	1
18:00–20:00	216 (56.7%)	1	108 (40.0%)	1	10:00–12:00	48 (11.1%)	7	66 (23.8%)	3
20:00–22:00	104 (27.3%)	2	8 (3.0%)	4	12:00–14:00	37 (8.5%)	8	56 (20.2%)	5
					14:00–16:00	89 (20.6%)	3	67 (24.2%)	2
					16:00–18:00	137 (31.6%)	2	80 (28.9%)	1
					18:00–20:00	208 (48.0%)	1	44 (15.9%)	6
					After 20:00	82 (18.9%)	4	10 (3.6%)	7

**Table 6:** Variables Predicting MPA Frequency: Results from Ordinal Logistic Regression Models.

Variables		Model 1 Combined Samples	Model 2 Urban Samples	Model 3 Rural Samples
		OR (95%CI)	OR (95%CI)	OR (95%CI)
Perceived Availability of PA Time		1.347 (1.162−1.562) ***	1.372 (1.138−1.654) **	1.368 (1.064−1.756) *
Perceived Availability of PA Space		1.756 (1.495−2.065) ***	1.701 (1.409−2.054) ***	2.387 (1.817−3.139) ***
Screen Time	Less than 30 min	3.991 (1.723−9.235) ***		
	0.5−1 h	3.074 (1.346−7.015) **		
	1−2 h	2.835 (1.220−6.593) *		
	2−3 h	1.974 (0.798−4.884)		
	More than 3 h	1.00		

*** Significance at 0.001, ** at 0.01, and * at 0.05 levels.

**Table 7:** Variables Predicting VPA frequency: Results from Ordinal Logistic Regression Models.

Variables		Model 1 Combined Samples	Model 2 Urban Samples	Model 3 Rural Samples
		OR (95%CI)	OR (95%CI)	OR (95%CI)
Perceived Availability of PA Time		1.160 (1.003−1.340) *		
Perceived Availability of PA Space		1.728 (1.474–2.026) ***	1.581 (1.347–1.857) ***	2.445 (1.931–3.096) ***
Screen Time	Less than 30 min	2.542 (1.104–5.859) *		3.216 (1.168–8.855) *
	0.5–1 h	1.876 (0.823–4.272)		2.038 (0.727–3.238)
	1–2 h	1.606 (0.694–3.717)		1.147 (0.406–3.238)
	2–3 h	1.241 (0.502–3.071)		1.287 (0.356–4.660)
	More than 3 h	1.00		1.00
Educational Level of Parents	Junior high school or less	1.799 (1.177–2.748) **		
	High school	1.284 (0.829–1.986)		
	Bachelor’s degree	0.971 (0.650–1.452)		
	Graduate degree	1.00		
Parents’ PA Habits	No PA habits		0.540 (0.314–0.929) *	
	PA habits		1.00	

*** Significance at 0.001, ** at 0.01, and * at 0.05 levels.

**Table 8:** Variables Predicting PFI: Results from Multiple Linear Regression Models.

Variables		Model 1 Combined (*n* = 696) (97.3%)		Model 2 Urban (*n* = 425) (97.0%)		Model 3 Rural (*n* = 272) (98.2%)	
		B (S.E.)	Beta	B (S.E.)	Beta	B (S.E.)	Beta
Weekly MVPA duration		0.016 *** (0.001)	0.403	0.016 *** (0.002)	0.392	0.015 *** (0.001)	0.542
Screen Time	more than 3 h	–3.468 ** (1.077)	–0.093			−2.084 * (0.931)	−0.099
	2–3 h	–2.354 ** (0.736)	–0.097				
	1–2 h	–1.293 * (0.551)	–0.073				
	0.5–1 h	–1.353 ** (0.433)	–0.095				
Perceived Availability of PA Space		0.921 *** (0.166)	0.178	1.354 *** (0.213)	0.275	1.136 *** (0.203)	0.266
Geographic Location: Urban		–5.119 *** (0.380)	–0.374				
Constant		74.020 *** (0.772)		66.586 *** (0.700)		72.540 *** (0.756)	
Adjusted R^2^		0.510		0.299		0.522	
R^2^		0.515		0.302		0.527	
F Statistic		104.243 *** (df = 7;688)		91.448 *** (df = 2;422)		99.497 *** (df = 3;268)	

Note: *** Significance at 0.001, ** at 0.01, and * at 0.05 levels; Reference categories: Screen time is less than 30 min; Geographic location is rural.

## Data Availability

The data are not publicly available due to the consent forms signed by participants indicating that the data would be available only to the team of investigators.

## References

[1] Dong Y., Jan C., Zou Z., Dong B., Hu P., Ma Y., Yang Z., Wang X., Li Y., Gao D. (2020). Comprehensive physical fitness and high blood pressure in children and adolescents: A national cross-sectional survey in China. J. Sci. Med. Sport.

[2] Ortega F.B., Ruiz J.R., Castillo M.J., Sjöström M. (2007). Physical fitness in childhood and adolescence: A powerful marker of health. Int. J. Obes..

[3] Ao D., Wu F., Yun C.-F., Zheng X.-Y. (2019). Trends in Physical Fitness among 12-Year-Old Children in Urban and Rural Areas During the Social Transformation Period in China. J. Adolesc. Health.

[4] Zaqout M., Vyncke K., Moreno L.A., De Miguel-Etayo P., Lauria F., Molnar D., Lissner L., Hunsberger M., Veidebaum T., Tornaritis M. (2016). Determinant factors of physical fitness in European children. Int. J. Public Health.

[5] Beltran-Valls M.R., Adelantado-Renau M., Moliner-Urdiales D. (2020). Reallocating time spent in physical activity intensities: Longitudinal associations with physical fitness (DADOS study). J. Sci. Med. Sport.

[6] Brown H., Pearson N., Braithwaite R., Brown W., Biddle S. (2012). Physical activity interventions and depression in children and adolescents: A systematic review and meta-analysis. J. Sci. Med. Sport.

[7] Carson V., Hunter S., Kuzik N., Gray C.E., Poitras V.J., Chaput J.-P., Saunders T.J., Katzmarzyk P., Okely A., Gorber S.C. (2016). Systematic review of sedentary behaviour and health indicators in school-aged children and youth: An update. Appl. Physiol. Nutr. Metab..

[8] Philbrook L.E., El-Sheikh M. (2016). Associations between neighborhood context, physical activity, and sleep in adolescents. Sleep Health.

[9] Riso E.-M., Toplaan L., Viira P., Vaiksaar S., Jurimae J. (2019). Physical fitness and physical activity of 6–7-year-old children according to weight status and sports participation. PLoS ONE.

[10] Guthold R., Stevens G.A., Riley L.M., Bull F.C. (2019). Global trends in insufficient physical activity among adolescents: A pooled analysis of 298 population-based surveys with 1·6 million participants. Lancet Child Adolesc. Health.

[11] Miao J., Wu X. (2016). Urbanization, socioeconomic status and health disparity in China. Health Place.

[12] Chen P. (2017). Physical activity, physical fitness, and body mass index in the Chinese child and adolescent populations: An update from the 2016 Physical Activity and Fitness in China—The Youth Study. J. Sport Health Sci..

[13] Aubert S., Barnes J.D., Abdeta C., Abi Nader P., Adeniyi A.F., Aguilar-Farias N., Tenesaca D.S.A., Bhawra J., Brazo-Sayavera J., Cardon G. (2018). Global Matrix 3.0 Physical Activity Report Card Grades for Children and Youth: Results and Analysis From 49 Countries. J. Phys. Act. Health.

[14] Si Q., Cardinal B.J. (2017). The health impact of air pollution and outdoor physical activity on children and adolescents in mainland China. J. Pediatr..

[15] Roberts J.D., Rodkey L., Ray R., Knight B., Saelens B.E. (2017). Electronic media time and sedentary behaviors in children: Findings from the built environment and active play study in the Washington DC area. Prev. Med. Rep..

[16] Liu Y., Tang Y., Cao Z.-B., Zhuang J., Zhu Z., Wu X.-P., Wang L.-J., Cai Y.-J., Zhang J.-L., Chen P.-J. (2019). Results from the China 2018 Report Card on physical activity for children and youth. J. Exerc. Sci. Fit..

[17] Zhu Z., Yang Y., Kong Z., Zhang Y., Zhuang J. (2017). Prevalence of physical fitness in Chinese school-aged children: Findings from the 2016 Physical Activity and Fitness in China—The Youth Study. J. Sport Health Sci..

[18] Shi Z., Lien N., Kumar B.N., Holmboe-Ottesen G. (2006). Physical activity and associated socio-demographic factors among school adolescents in Jiangsu Province, China. Prev. Med..

[19] Sheu-Jen H., Wen-Chi H., Patricia A.S., Jackson P.W. (2010). Neighborhood environment and physical activity among Urban and Rural Schoolchildren in Taiwan. Health Place.

[20] Akpınar A. (2020). Investigating the barriers preventing adolescents from physical activities in urban green spaces. Urban For. Urban Green..

[21] Bai H., Stanis S.A.W., Kaczynski A.T., Besenyi G. (2013). Perceptions of Neighborhood Park Quality: Associations with Physical Activity and Body Mass Index. Ann. Behav. Med..

[22] Cui Z., Hardy L.L., Dibley M.J., Bauman A. (2011). Temporal trends and recent correlates in sedentary behaviours in Chinese children. Int. J. Behav. Nutr. Phys. Act..

[23] Sandercock G.R., Ogunleye A., Voss C. (2012). Screen Time and Physical Activity in Youth: Thief of Time or Lifestyle Choice?. J. Phys. Act. Health.

[24] Khan A., Mandic S., Uddin R. (2021). Association of active school commuting with physical activity and sedentary behaviour among adolescents: A global perspective from 80 countries. J. Sci. Med. Sport.

[25] Jiménez-Pavón D., Fernández-Alvira J., Velde S., Brug J., Bere E., Jan N., Kovacs E., Androutsos O., Manios Y., Bourdeaudhuij I.D. (2012). Associations of parental education and parental physical activity (PA) with children’s PA: The ENERGY cross-sectional study. Prev. Med..

[26] Akpinar A., Cankurt M. (2016). Parental influence on children’s physical activity in urban green spaces. İstanbul Üniversitesi Orman Fakültesi Derg..

[27] Malina R.M., Katzmarzyk P.T. (2006). Physical activity and fitness in an international growth standard for preadolescent and adolescent children. Food Nutr. Bull..

[28] Salmon J., Veitch J., Abbott G., Chinapaw M., Brug J.J., Tevelde S.J., Cleland V., Hume C., Crawford D., Ball K. (2013). Are associations between the perceived home and neighbourhood environment and children′s physical activity and sedentary behaviour moderated by urban/rural location?. Health Place.

[29] Ujevic T., Sporis G., Milanovic Z., Pantelic S., Neljak B. (2013). Differences between health-related physical fitness profiles of croatian children in urban and rural areas. Coll. Antropol..

[30] Moore J.B., Brinkley J., Crawford T.W., Evenson K.R., Brownson R.C. (2013). Association of the built environment with physical activity and adiposity in rural and urban youth. Prev. Med..

[31] He X.F. (2019). The Foundation of a Great Nation: Problems of China’s Rural Revitalization.

[32] Lu W.Y., Liang W., Sun L., Luo F., Sun X.B., Wang X.Y. (2010). Research on current situation, problems and its countermeasures about public sport service supply in rural areas of western region under the circumstance of constructing new rural areas. China Sport Sci..

[33] Luo J., Zheng B., Lu W.Y., Liu S.F. (2011). Research on current health situation and constraints factors of farmers in west region of China. China Sport Sci..

[34] Li Q.Y., Zhong S.Y. (2016). Spatial inequality and distributional dynamics of sports resource allocation in China. China Sport Sci..

[35] Lizana P.A., Paula C.-V., Araya L., Aguilera F., Mora M. (2016). Obesity, Body Fat Distribution, and Physical Activity in School-age Children: An Urban and Rural Comparison in Valparaíso, Chile. Biomed. Environ. Sci..

[36] Rivera-Ochoa M., Brazo-Sayavera J., Vizmanos-Lamotte B., Mañas A., López-Taylor J.R., González-Gross M., Guadalupe-Grau A. (2020). Health-Related Factors in Rural and Urban Mexican Adolescents from the State of Jalisco: The HELENA-MEX Study. Int. J. Environ. Res. Public Health.

[37] Fan X., Zhu Z., Zhuang J., Liu Y., Tang Y., Chen P., Cao Z.B. (2019). Gender and age differences in the association between living arrangement and physical activity levels among youth aged 9–19 years in Shanghai, China: A cross-sectional questionnaire study. BMC Public Health.

[38] Bureau of Statistics of Shannxi Province. http://tjj.shaanxi.gov.cn/upload/n2020/zk/indexch.htm.

[39] Bureau of Statistics of Shannxi Province. http://tjj.shaanxi.gov.cn/tjsj/ndsj/tjgb/qs_444/202003/t20200319_1617383.html.

[40] Yoon J., Lee C. (2019). Neighborhood outdoor play of White and Non-White Hispanic children: Cultural differences and environmental disparities. Landsc. Urban Plan..

[41] Ministry of Education of the People’s Republic of China http://www.moe.gov.cn/s78/A17/twys_left/moe_938/moe_792/s3273/201407/t20140708_171692.html.

[42] Wu C.-L., Chang C.-K. (2019). Results from the Chinese Taipei (Taiwan) 2018 Report Card on physical activity for children and youth. J. Exerc. Sci. Fit..

[43] Prins R.G., Oenema A., Van Der Horst K., Brug J. (2009). Objective and perceived availability of physical activity opportunities: Differences in associations with physical activity behavior among urban adolescents. Int. J. Behav. Nutr. Phys. Act..

[44] Scott M.M., Evenson K.R., Cohen D.A., Cox C.E. (2007). Comparing Perceived and Objectively Measured Access to Recreational Facilities as Predictors of Physical Activity in Adolescent Girls. J. Hered..

[45] Maddison R., Hoorn S.V., Jiang Y., Ni Mhurchu C., Exeter D., Dorey E., Bullen C., Utter J., Schaaf D., Turley M. (2009). The environment and physical activity: The influence of psychosocial, perceived and built environmental factors. Int. J. Behav. Nutr. Phys. Act..

[46] Ball K., Jeffery R.W., Crawford D., Roberts R., Salmon J., Timperio A.F. (2008). Mismatch between perceived and objective measures of physical activity environments. Prev. Med..

[47] Keane E., Li X., Harrington J.M., Fitzgerald A.P., Perry I., Kearney P. (2017). Physical Activity, Sedentary Behavior and the Risk of Overweight and Obesity in School-Aged Children. Pediatr. Exerc. Sci..

[48] O’Connor T.M., Chen T.-A., Baranowski J., Thompson D., Baranowski T. (2013). Physical activity and screen-media-related parenting practices have different associations with children’s objectively measured physical activity. Child. Obes..

[49] Sandercock G.R.H., Ogunleye A.A. (2012). Screen time and passive school travel as independent predictors of cardiorespiratory fitness in youth. Prev. Med..

[50] Maddison R., Marsh S., Foley L., Epstein L.H., Olds T., Dewes O., Heke I., Carter K., Jiang Y., Mhurchu C.N. (2014). Screen-Time Weight-loss Intervention Targeting Children at Home (SWITCH): A randomized controlled trial. Int. J. Behav. Nutr. Phys. Act..

[51] Ferrari G.L.D., Pires C., Sole D., Matsudo V., Katzmarzyk P.T., Fisberg M. (2019). Factors associated with objectively measured total sedentary time and screen time in children aged 9–11 years. J. Pediatr..

[52] Sun J.T., Meng A., Bo Y.X., Hua L.G., Bo W.D. (2018). Prevalence and related factors for myopia in school-aged children in Qingdao. J. Ophthalmol..

[53] Yang-Huang J.W., van Grieken A., Wang L., Jaddoe V.W.V., Jansen W., Raat H. (2018). Ethnic background and children’s television viewing trajectories: The generation R study. PLoS ONE.

[54] Webster E.K., Martin C.K., Staiano A.E. (2019). Fundamental motor skills, screen-time, and physical activity in preschoolers. J. Sport Health Sci..

[55] China National Children’s Center https://baijiahao.baidu.com/s?id=1642381232600450024&wfr=spider&for=pc2019.

[56] Ministry of Education of the People’s Republic of China http://www.moe.gov.cn/jyb_xwfb/gzdt_gzdt/s5987/201808/t20180830_346673.html.

[57] Li M., Dibley M.J., Sibbritt D., Yan H. (2008). Factors associated with adolescents’ overweight and obesity at community, school and household levels in Xi’an City, China: Results of hierarchical analysis. Eur. J. Clin. Nutr..

[58] Cheng T.O. (2004). Obesity in Chinese children. J. Roy. Soc. Med..

[59] Dozier S.G.H., Schroeder K., Lee J., Fulkerson J.A., Kubik M.Y. (2020). The association between parents and children meeting physical activity guidelines. J. Pediatr. Nurs..

[60] Yan H., Chen J.D., Huang J. (2019). School bullying among left-behind children: The efficacy of art therapy on reducing bullying victimization. Front. Psychiatry.

[61] Mota J., Santos R., Pereira M., Teixeira L., Santos M.P. (2011). Perceived neighborhood environmental characteristics and physical activity according to socioeconomic status in adolescent girls. Ann. Hum. Biol..

[62] Tudor-Locke C., Ainsworth B.E., Adair L.S., Popkin B.M. (2003). Objective physical activity of Filipino youth stratified for commuting mode to school. Med. Sci. Sports Exerc..

[63] Sun Y., Liu Y., Tao F.B. (2015). Associations between active commuting to school, body fat, and mental well-being: Population-based, cross-sectional study in China. J. Adolesc. Health.

[64] Fan X., Cao Z.B. (2017). Physical activity among Chinese school-aged children: National prevalence estimates from the 2016 Physical Activity and Fitness in China—The Youth Study. J. Sport Health Sci..

[65] Wang H.X., Han Z.J. (2009). Study on the county-level city in China. J. Politics Law.

